# Titanium Dioxide Nanoparticles Aggravated the Developmental Neurotoxicity of Ammonia Nitrogen on Zebrafish Embryos

**DOI:** 10.3390/toxics13121031

**Published:** 2025-11-28

**Authors:** Minglei Lyu, Jiaqian Yu, Qing Yang, Yi Shen, Haoling Liu, Xuanjie Wang, Xiaolin Liu, Fang Shi, Xi Zou, Jinmiao Zha, Guangyu Li, Xufa Ma

**Affiliations:** 1 College of Fisheries, Huazhong Agricultural University, Wuhan 430070, China; 2 Key Laboratory of Ecological Impacts of Hydraulic Projects and Restoration of Aquatic Ecosystem, Ministry of Water Resources and Chinese Academy of Sciences, Wuhan 430079, China; 3 National Engineering Research Center of Industrial Wastewater Detoxication and Resource Recovery, Research Center for Eco-Environmental Sciences, Chinese Academy of Sciences, Beijing 100085, China

**Keywords:** ammonia toxicity, n-TiO_2_, developmental neurotoxicity, combined pollution

## Abstract

Total ammonia nitrogen (TAN) is a common and potent neurotoxic pollutant in aquatic environments. Due to their strong adsorption capacity, titanium dioxide nanoparticles (n-TiO_2_), a widely used engineered material, can induce combined toxicity with multiple pollutants. However, the combined neurotoxicity of n-TiO_2_ and TAN and its underlying mechanisms remain unclear. In this study, zebrafish embryos were exposed to TAN (0, 0.1, 1, 10 mg/L) and n-TiO_2_ (100 µg/L) individually or in combination for 120 h. The results indicated that co-exposure to n-TiO_2_ and TAN significantly increased the bioaccumulation of TAN in zebrafish embryos compared to TAN alone. Consequently, this led to exacerbated neurotoxicity, manifested as developmental impairments and abnormal motor behavior. Mechanistic investigations revealed that the co-exposure aggravated developmental neurotoxicity by triggering neuronal apoptosis and oxidative stress, disrupting the cholinergic and dopaminergic systems, and impairing neural and retinal development. Transcriptomic analysis further indicated that the co-exposure predominantly perturbed neurodevelopment, oxidative stress, and apoptosis. In conclusion, this study confirms that n-TiO_2_ significantly amplifies TAN-induced neurodevelopmental toxicity by promoting its bioaccumulation and synergistically disrupting multiple neurophysiological processes. These findings provide crucial scientific evidence for assessing the combined ecological risks of nanomaterials and conventional pollutants.

## 1. Introduction

Total ammonia nitrogen (TAN) is a pollutant of global concern due to its ubiquity in surface waters and high toxicity to aquatic life. TAN is primarily derived from excreta in agriculture, animal husbandry, industry, and other sectors [1,2]. In water, TAN exists mainly in the ionic form as ammonium (NH_4_^+^) and the non-ionic form as ammonia (NH_3_) [3]. Of the two forms, NH_3_ is more toxic because its electrical neutrality and high lipid solubility facilitate diffusion across cell membranes, leading to intracellular ammonia accumulation and toxicity [4]. In aquaculture water bodies, TAN concentrations often exceed 10 mg/L [5]. TAN levels in some polluted rivers have reached concentrations comparable to aquaculture water standards. Wang et al. [6] reported that both TAN and non-ionic ammonia in the Liaohe River showed an increasing trend between 2013 and 2018, with the acute water quality benchmark for TAN determined to be 16.86 mg/L. High concentrations of TAN can cause abnormalities in neurotransmitter metabolism, thereby overactivating NMDA-type glutamate receptors (NMDARs) and impairing central nervous system function [7,8] Previous studies have shown that TAN exposure induces abnormal social behavior in zebrafish, which may compromise individual fitness and population survival competitiveness [9]. However, most current research on ammonia neurotoxicity has focused on mammals or adult fish, leaving the mechanisms of developmental neurotoxicity in zebrafish embryos poorly understood.

As one of the most widely produced engineered nanomaterials (ENMs) globally, titanium dioxide nanoparticles (n-TiO_2_) are extensively used in consumer products such as coatings, sunscreens, and cosmetics [10,11] Their large-scale production and use result in substantial and continuous environmental release, raising public concern over the associated environmental risks [12,13]. The adverse effects of n-TiO_2_ on aquatic environments primarily stem from its unique physicochemical properties, particularly its large specific surface area and strong adsorption capacity. These characteristics enable n-TiO_2_ to act as a carrier for adsorbing other pollutants, thereby influencing the interaction between contaminants and organisms [14,15]. Evidence indicates that the carrier effect of n-TiO_2_ can exacerbate the neurotoxicity of pollutants. For example, n-TiO_2_ can form complexes with microcystin-LR (MC-LR), enhancing oxidative stress in the zebrafish brain and aggravating MCLR-induced brain tissue damage [16]. Furthermore, n-TiO_2_ can promote the bioaccumulation of triphenyl phosphate in zebrafish, leading to alterations in locomotor behavior by inhibiting neuronal development, damaging axons of secondary motor neurons, and reducing serotonin (5-HT) content [17]. Currently, the studies on the combined neurotoxicity of n-TiO_2_ with pollutants mainly focus on its association with organic pollutants. The combined neurotoxic effects and mechanisms of n-TiO_2_ with inorganic pollutants deserve further exploration.

In recent years, n-TiO_2_ has been widely applied and inevitably entered aquatic environments to interact with TAN [18,19]. However, whether n-TiO_2_ exacerbates the neurotoxic effects of TAN remains elusive. In this study, zebrafish embryos were selected as the model organism due to their significant advantages in developmental biology and toxicological research. Their external fertilization and rapid, transparent developmental process allow for direct, non-invasive, and real-time observation of morphological abnormalities and organogenesis [20]. Zebrafish embryos were used as the model organisms and exposed to TAN (0, 0.1, 1, 10 mg/L) with or without n-TiO_2_ (100 µg/L) for 120 h post-fertilization (hpf). The neurotoxicity mechanisms of n-TiO_2_ and TAN were thoroughly investigated from multiple perspectives, including behavioral studies, histopathology, neurotransmitter levels, enzyme activity, gene expression, and transcriptomic analysis. The findings of this study could provide a theoretical basis for early warning of aquatic ecological risks posed by combined pollution from ENMs and traditional pollutants.

## 2. Materials and Methods

### 2.1. Chemicals

NH_4_Cl (purity > 99.5%) was purchased from China National Pharmaceutical Chemical Reagent Co., Ltd. (Shanghai, China). n-TiO_2_ (purity > 99%, particle size < 100 nm) was purchased from Beijing Northern Weiye Metrology Technology Research Institute Co., Ltd. (Beijing, China). All other chemicals used in this experiment were of analytical grade.

### 2.2. Characterization of n-TiO_2_ Suspension

The n-TiO_2_ stock suspension was added to ultrapure water to prepare a 100 µg/L solution. After vortex mixing, the suspension was subjected to ultrasonic treatment for 20 min to prepare a fresh n-TiO_2_ suspension for testing. The particle size distribution of n-TiO_2_ was observed using transmission electron microscopy (TEM; HT-7700 model, Hitachi Ltd., Tokyo, Japan). The zeta potential of n-TiO_2_ was analyzed using a Zetasizer instrument (Malvern, UK).

### 2.3. Exposure of Embryos

Zebrafish embryos (AB strain) were obtained from the Institute of Hydrobiology, Chinese Academy of Sciences (Wuhan, China). Normally developing embryos were selected at 0.5–1.0 h post-fertilization (hpf) for acute exposure experiments. Embryos were randomly allocated to experimental groups using a random number table to ensure baseline consistency between groups. Eight exposure groups were established: control group (without TAN and n-TiO_2_), TAN alone groups (0.1, 1, and 10 mg/L), n-TiO_2_ alone group (100 µg/L), and n-TiO_2_ combined with TAN groups (0.1, 1, 10 mg/L). Each group included three independent biological replicates. Each replicate consisted of 1200 embryos distributed across six standard 90 mm polystyrene culture dishes (200 embryos per dish). During transfer to culture dishes, embryos were evenly distributed across the bottom of each dish using wide-bore pipettes with slow rotational movements to avoid local aggregation and ensure spatial distribution consistency of 200 embryos per dish. Exposure experiments were conducted using standard 90 mm polystyrene Petri dishes. Each dish was filled to approximately two-thirds of its capacity with exposure solution, corresponding to a working volume of 30 mL. The water volume per embryo was approximately 0.15 mL. Throughout the exposure period, dishes were spatially arranged in the incubator using a randomized block design and maintained with loose-fitting lids to ensure adequate gas exchange while minimizing evaporation. After 120 h, larvae were collected on ice, rapidly frozen in liquid nitrogen in order to instantaneously terminate all biochemical reactions and completely preserve the molecular profile of the larvae at the time of sampling, before storage at −80 °C for subsequent analysis. Acute endpoint metrics (mortality, malformation rate, body length) were recorded from at least three replicate groups. Heart rate (beats/10 s) was counted under a stereomicroscope (Leica M205 FA, 8× magnification). To maintain stable TAN and n-TiO_2_ concentrations, all experimental solutions were replaced daily with fresh solutions of equivalent concentrations. Dead larvae were removed from culture dishes to prevent bacterial contamination of the medium. This study was approved by the Institutional Animal Care and Use Committee (IACUC, Wuhan, China) of Huazhong Agricultural University.

### 2.4. TAN Levels in Zebrafish Larvae

Detection of TAN content in zebrafish larvae was performed using an ammonia assay kit (Jiangsu AidiSheng Biotechnology Co., Ltd., Nanjing, China) according to the manufacturer’s instructions. This method utilizes the reaction of TAN with hypochlorite and phenol in a strongly alkaline environment to generate the water-soluble dye indophenol blue, which exhibits a characteristic absorption peak at 630 nm. The absorbance value is directly proportional to the TAN concentration, enabling the determination of total TAN content. Specifically, collected zebrafish larvae (100 per tube) were mixed with TAN extraction solution at a mass (g) to volume (mL) ratio of 1:9. The mixture was homogenized on ice for 5 min, and then centrifuged at 12,000 rpm at room temperature for 10 min. The supernatant from all treatment groups (n = 3) was collected for TAN content determination.

### 2.5. Detection of Locomotor Behaviour in Zebrafish Larvae

The locomotor behavior was quantified using a video tracking system (ViewPoint LifeSciences, Montreal, QC, Canada) in zebrafish larvae at 120 h post-fertilization (hpf). Larvae were individually placed in 24-well plates with 2 mL of purified water per well. Each experimental group utilized two 24-well plates (see Figure S1), with three biological replicates per group, totaling six culture plates. To eliminate inter-plate variation, locomotor behavior data were collected simultaneously from all six plates. Larvae were acclimated for 30 min prior to swimming speed monitoring, with the 24-well plates maintained at 28 °C. Swimming speed was monitored under continuous visible light and in response to dark-to-light transitions (5 min light, 5 min dark, 5 min light, 5 min dark). Data were collected every 30 s, including distance traveled during dark and light periods, average speed, and movement duration. Each experiment was repeated four times. Data were further analyzed using customized OpenOffice.org 2.4 software.

### 2.6. Oxidative Stress Analysis

Detection of malondialdehyde (MDA) and glutathione (GSH) levels, as well as superoxide dismutase (SOD) and glutathione peroxidase (Gpx) activity, was performed using assay kits purchased from Jiangsu Addison Biotechnology Co., Ltd. (Yancheng, China). In brief, the collected zebrafish larvae (100 per tube) were added to 1 mL extraction buffer and homogenized on ice. The mixture was centrifuged at 4 °C and 12,000 rpm for 15 min. The supernatant was collected and assayed following the manufacturer’s instructions.

### 2.7. Acridine Orange (AO) Staining

At 120 hpf, 10 zebrafish larvae per group were randomly selected with 3 replicates. The larvae were rinsed three times with PBST, then placed in a 5 µg/mL AO solution for 30 min in the dark. The AO solution was discarded, and the larvae were rinsed three times with phosphate-buffered saline with Tween-20 (PBST) to thoroughly remove surface staining before observation under a fluorescence stereomicroscope.

### 2.8. Detection of Caspase Enzyme Activity

Caspase 3, Caspase 8, and Caspase 9 were detected using kits purchased from Nanjing Jiancheng Bio-Tech Co., Ltd. (Nanjing, China). The lysis buffer working solution and 2× reaction buffer were prepared. After processing zebrafish larvae samples, the supernatant was centrifuged and collected. The reagents were then added according to the specific reaction system. Incubation was performed at 37 °C, and the absorbance was measured at 405 nm using an enzyme-linked immunosorbent assay (ELISA) reader (Molecular Devices Shanghai Corporation, Shanghai, China).

### 2.9. Detection of Dopamine Levels

Dopamine (DA) levels in zebrafish larvae were measured using an ELISA method developed by Nanjing Jiancheng Bioengineering Institute (Nanjing, China). Specifically, 300 larvae were randomly collected from each group (n = 3). Samples were weighed and diluted with PBS (pH 7.4) at a 1:9 (*w*/*v*) ratio. Specimens were thoroughly homogenized using a homogenizer and then centrifuged at 3000 rpm for 20 min at 4 °C. The supernatant was collected and analyzed according to the manufacturer’s instructions.

### 2.10. Determination of AChE Activity

For acetylcholinesterase (AChE) activity assays, 100 larvae were randomly selected (n = 3). On ice, 0.9% physiological saline was added at a 1:9 (*w*/*v*) ratio, followed by centrifugation at 3000 rpm for 10 min at 4 °C. The supernatant was transferred to a fresh tube for protein content and AChE activity assays. Kits for total protein content and AChE activity detection were purchased from Nanjing Jiancheng Bioengineering Institute (Nanjing, China).

### 2.11. Gene Expression

Zebrafish larvae from each group were placed in enzyme-free centrifuge tubes (30 larvae per tube, n = 3). Larval tissues were lysed using the Trizol method to extract total RNA. RNA purity was assessed, and samples with an A260/A280 ratio between 1.8 and 2.1 were selected. Reverse transcription was performed using a reverse transcription kit (TaKaRa, PrimeScript^®^, Kusatsu, Japan) according to the manufacturer’s instructions to convert purified RNA into cDNA. Gene expression levels related to neural and retinal development were analyzed using a quantitative PCR kit (TaKaRa, SYBR Green^®^, Kusatsu, Japan). Primer sequences for genes related to neural and retinal development were designed using NCBI (https://www.ncbi.nlm.nih.gov/). Primer sequences are presented in Table S1. Relative mRNA expression levels were determined using the relative Ct method (2^–ΔΔCt^).

### 2.12. Transcriptomics Sequencing

Approximately 100 larvae were randomly selected from each group and transferred to Eppendorf (EP) tubes (n = 3). After 3 washes with PBS, moisture was removed by pipetting, followed by rapid freezing in liquid nitrogen and storage at −80 °C. Transcriptome sequencing was performed using the DNBSEQ platform (Meiji Bio, Shanghai, China), yielding a total of 12 samples. The full transcriptome sequencing (RNA-Seq) workflow comprised total RNA extraction and quality control, sequencing library preparation, and machine sequencing. Total RNA was extracted using the Trizol method and quality-assessed with an Agilent 2100 Bioanalyzer (Agilent Technologies, Inc., Santa Clara, CA, USA). mRNA enrichment was performed on the total RNA. The obtained RNA was fragmented using a fragmentation reagent, reverse transcribed into cDNA, and purified into double-stranded DNA. The synthesized double-stranded DNA was end-blunted and repaired, then ligated with specific adapters. The ligation products were amplified via PCR using specific primers. PCR products were denatured into single strands, which were then circularized into single-stranded circular DNA libraries using bridge primers. The constructed libraries underwent quality control, and those meeting standards proceeded to sequencing.

### 2.13. Statistical Analyses

All data were analyzed with SPSS 27.0 (IBM SPSS Statistics, Armonk, NY, USA) and expressed as mean standard error (mean ± SEM). The data’s adherence to normal distribution was assessed with the Shapiro–Wilk test, while the homogeneity of variance was examined through Levene’s test. Tukey’s test and one-way analysis of variance (ANOVA) were utilized to assess the variations between the exposed and control groups. When homogeneity of variance was not satisfied, the independent samples *t*-test was used to analyze the differences between the two sets of data. Statistical significance was established for *p* values < 0.05.

## 3. Results

### 3.1. n-TiO_2_ Characterisation

**Figure 1:**
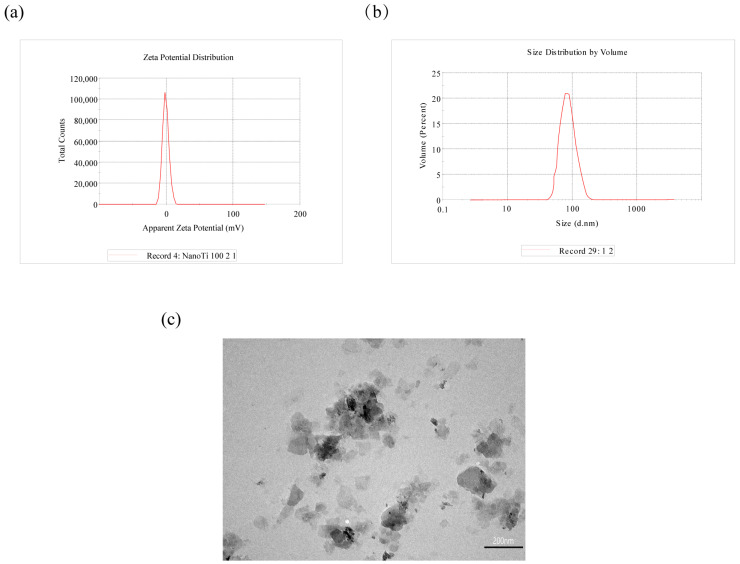
Characterization of n-TiO_2_: (**a**) zeta potential of n-TiO_2_; (**b**) particle size distribution in n-TiO_2_ exposure solution; (**c**) transmission electron microscopy image of n-TiO_2_.

The zeta potential of n-TiO_2_ in suspension was measured at −1.2 mV using a Zetasizer nanoparticle analyzer (Figure 1a), with an average particle size distribution of 99.8 nm in the n-TiO_2_ exposure solution (Figure 1b). Transmission electron microscopy (TEM) observations and images (Figure 1c) revealed that n-TiO_2_ existed in aqueous solution as primary particles and small aggregates.

### 3.2. Developmental Toxicity

**Figure 2:**
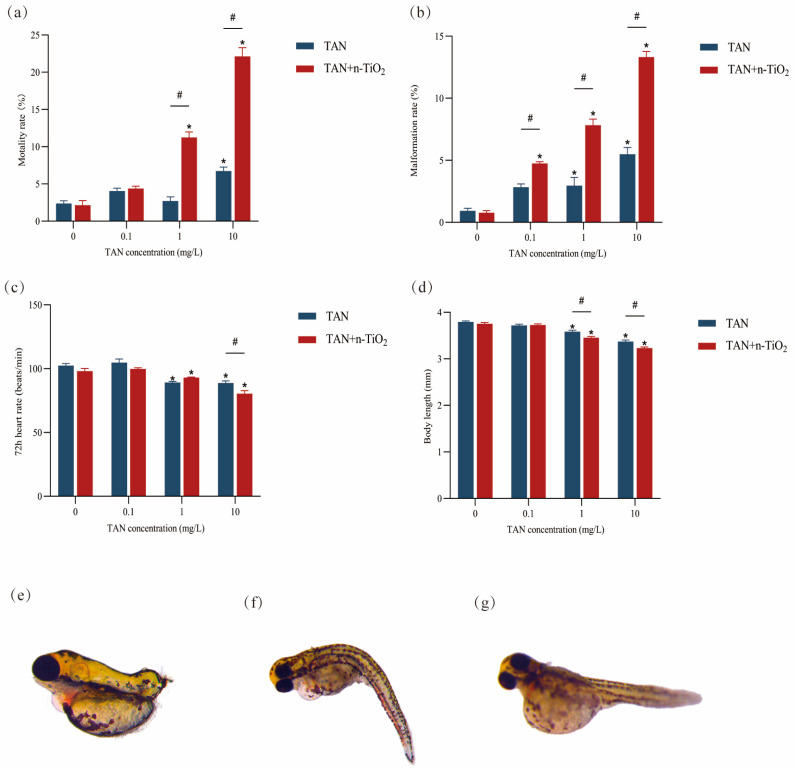
Zebrafish embryonic developmental endpoints and representative morphological malformations at 120 hpf: (**a**) mortality rate (%); (**b**) malformation rate (%); (**c**) heart rate (beats/min); (**d**) body length (mm); (**e**) representative image showing tail hypoplasia; (**f**) representative image showing spinal curvature; (**g**) representative image showing yolk sac edema. Data are presented as mean ± SEM (n = 3 independent experiments). Statistical significance was determined by one-way ANOVA with Tukey’s post hoc test. * indicates significant difference between control and exposed groups (*p* < 0.05); # indicates significant difference between TAN and n-TiO_2_ + TAN treatments (*p* < 0.05).

Statistical analysis was conducted on the mortality rate, malformation rate, heart rate, and body length of juvenile fish exposed to pollutants (Figure 2). Results indicated that exposure to n-TiO_2_ alone did not affect zebrafish development. Compared with the control group, mortality rates were significantly elevated in the 1 mg/L TAN + n-TiO_2_ group, the 10 mg/L TAN group, and the 10 mg/L TAN + n-TiO_2_ group (*p* < 0.05). Additionally, the malformation rate of zebrafish embryos significantly increased in the 1 mg/L TAN group, the 10 mg/L TAN group, and all combined exposure groups (*p* < 0.05). Regardless of exposure type, zebrafish larvae exhibited significantly reduced heart rate and body length in both the 1 mg/L TAN and 10 mg/L TAN groups (*p* < 0.05), with the decline being significantly greater in the high-concentration combined exposure group compared to the single-exposure group. Based on changes in toxicity endpoint indicators, n-TiO_2_ significantly exacerbated the adverse effects of TAN on zebrafish embryo development compared to TAN alone. Morphological observations were conducted on zebrafish larvae at 120 hpf. Larvae in the co-exposure groups exhibited a variety of severe malformations, including tail hypoplasia (Figure 2e), spinal curvature (Figure 2f), and yolk sac edema (Figure 2g).

### 3.3. Behavioural Detection

**Figure 3:**
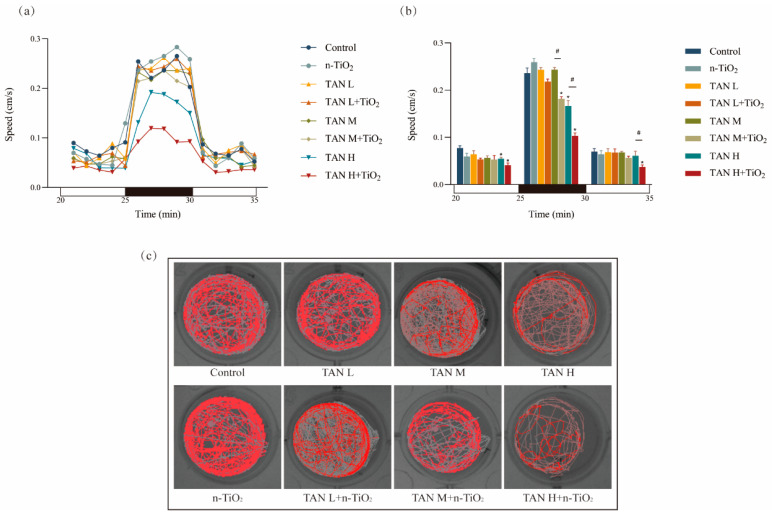
Locomotor behavior analysis of 120 hpf zebrafish larvae following 120 h exposure to TAN and n-TiO_2_: (**a**) mean swimming velocity (cm/s) recorded at 60-s intervals during light–dark transition stimulation. The horizontal axis shows dark phases (black bars, 25–30 min) and light phases (white bars, 20–25 min and 30–35 min); (**b**) average swimming speed (cm/s) calculated per 5 min photoperiod interval, summarizing locomotor activity during each light or dark phase; (**c**) representative movement trajectories of larvae during the dark phase (red curve); * indicates significant differences between the control and exposed groups (*p* < 0.05); # indicates significant difference between TAN and n-TiO_2_ + TAN treatments (*p* < 0.05). Note: TAN L: low-concentration TAN; TAN M: medium-concentration TAN; TAN H: high-concentration TAN.

Swimming behavior of zebrafish larvae was further assessed at 120 hpf using the DanioVision™ (Noldus, Beijing, China) behavior system, with results shown in Figure 3. Findings revealed that swimming activity during the light period was reduced compared to the dark period. Compared to the control group, all three experimental groups, 10 mg/L TAN alone, 1 mg/L TAN + n-TiO_2_, and 10 mg/L TAN + n-TiO_2_, exhibited delayed activity decline during the transition from dark to light. Average larval activity was quantified during each 5 min photoperiod interval (Figure 3b). During the dark period, the average swimming speed of larvae in the 10 mg/L TAN + n-TiO_2_ group was significantly reduced. Figure 3c illustrates the movement trajectories of zebrafish larvae during the photoperiod.

### 3.4. TAN Content in Zebrafish Larvae

**Figure 4:**
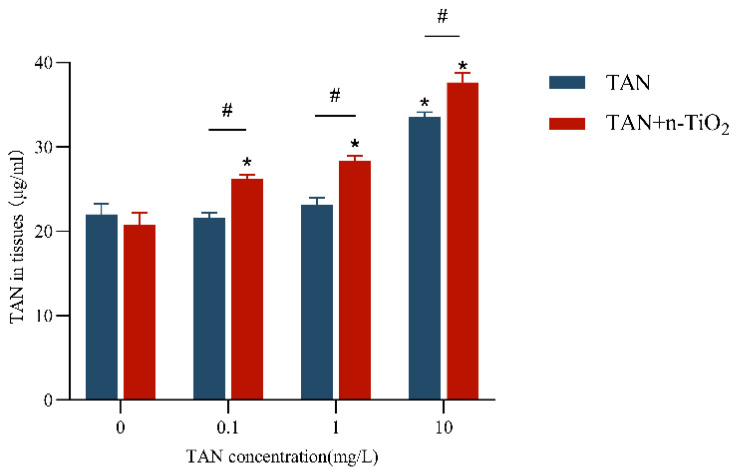
TAN content in zebrafish larvae following 120 h exposure. Quantification of total ammonia nitrogen accumulation in larval tissues after exposure to control, TAN alone (0.1, 1, 10 mg/L), n-TiO_2_ alone (100 µg/L), and co-exposure treatments. Data are presented as mean ± SEM (n = 3 biological replicates). * *p* < 0.05 vs. control group; # *p* < 0.05 between TAN alone and corresponding co-exposure groups.

TAN levels were measured in tissues of zebrafish larvae (Figure 4). The results showed that in the group exposed to TAN alone, TAN content in the high-concentration (10 mg/L) TAN exposure group was significantly elevated compared to the control group (*p* < 0.05). Sole n-TiO_2_ exposure had no significant effect on TAN metabolism. However, when n-TiO_2_ and TAN were co-exposed, TAN levels significantly increased (*p* < 0.05) in both the low-concentration TAN group (0.1 mg/L) and the high-concentration TAN group (10 mg/L) compared to the TAN-only group. This indicated that n-TiO_2_ enhanced TAN accumulation.

### 3.5. Measurement of Oxidative Stress

**Figure 5:**
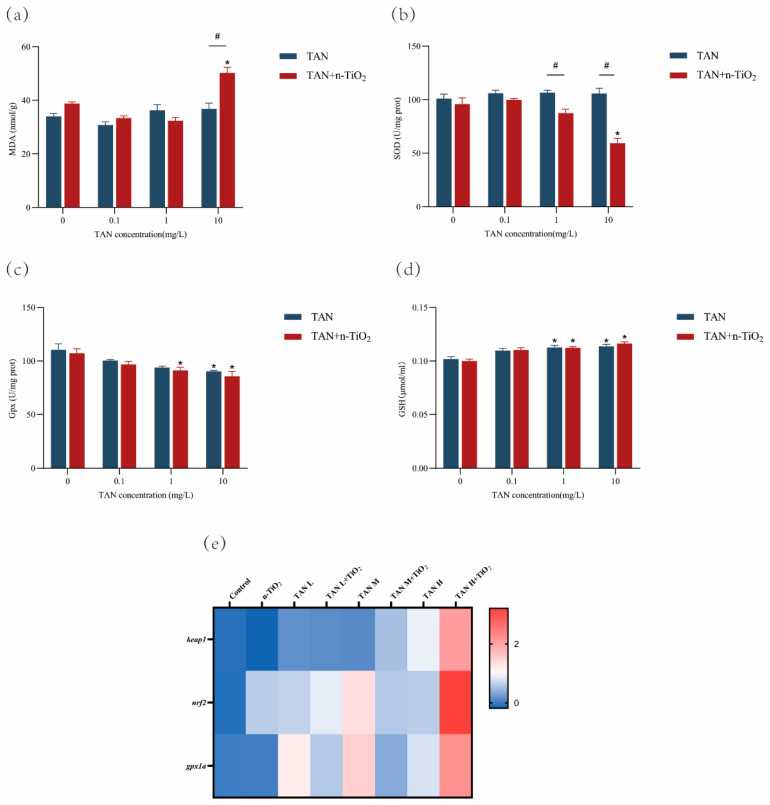
Oxidative stress markers in zebrafish larvae at 120 hpf following 120 h exposure: (**a**) MDA content; (**b**) SOD activity; (**c**) Gpx activity; (**d**) GSH content; (**e**) heatmap of oxidative stress-related gene expression levels, where values represent log_2_-transformed fold changes in gene expression. Data are presented as mean ± SEM (n = 3 biological replicates); * indicates significant differences between control and exposed groups (*p* < 0.05); # indicates significant differences between TAN and TAN + n-TiO_2_ treatment groups (*p* < 0.05).

Assessment of oxidative stress markers revealed (Figure 5) that compared with the control group, exposure to n-TiO_2_ alone (100 µg/L) had no significant effect on MDA content, GSH levels, SOD, and Gpx activity. In the TAN-exposed group alone, MDA content showed no significant change. GSH levels in the 1 mg/L and 10 mg/L groups were significantly higher than those in the control group (*p* < 0.05). Gpx activity exhibited a decreasing trend with increasing concentrations, with activity in the 10 mg/L group significantly lower than the control group. When co-exposed to n-TiO_2_ and TAN, oxidative stress damage was further exacerbated. MDA content in the 10 mg/L TAN + n-TiO_2_ group was significantly higher than in the 10 mg/L TAN alone group. SOD activity showed a marked reduction in the 10 mg/L TAN + n-TiO_2_ group compared to the 10 mg/L TAN alone group. These results indicated that TAN exposure induced oxidative stress in zebrafish larvae, and the presence of n-TiO_2_ enhanced this effect.

### 3.6. AO Staining

**Figure 6:**
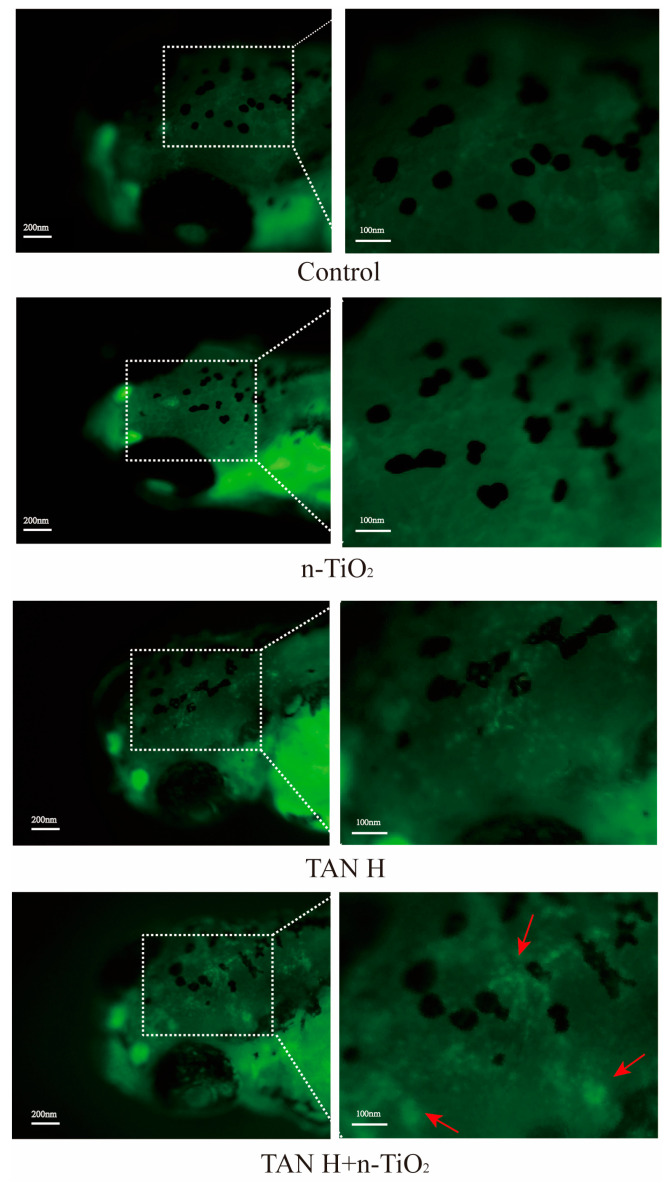
AO Staining in 120 hpf zebrafish larvae. Red arrows indicate distinct apoptotic cell regions, with prominent fluorescent signals observed in neural tissues, including the brain and spinal cord. Imaging parameters were maintained consistent (ISO 100, f/11, 1/5 s).

AO staining specifically labeled apoptotic cells (bright green fluorescence). Results are shown in Figure 6. The control group exhibited uniform green fluorescence overall, indicating normal cellular structure and nucleic acid distribution. The number of apoptotic cells in the n-TiO_2_ alone exposure group showed no significant difference compared to the control group. In the TAN alone exposure group, the number of apoptotic cells increased with rising concentration. The fluorescence intensity in neural tissues such as the brain and spinal cord was significantly enhanced in the 10 mg/L group. The combined exposure groups exhibited further increases in apoptotic cells. The fluorescence signal coverage was markedly expanded in the 10 mg/L TAN + n-TiO_2_ group, particularly in the midbrain and medulla regions, indicating that n-TiO_2_ exacerbated ammonia-induced neuronal apoptosis.

### 3.7. Result of Caspase Enzyme Activity

**Figure 7:**
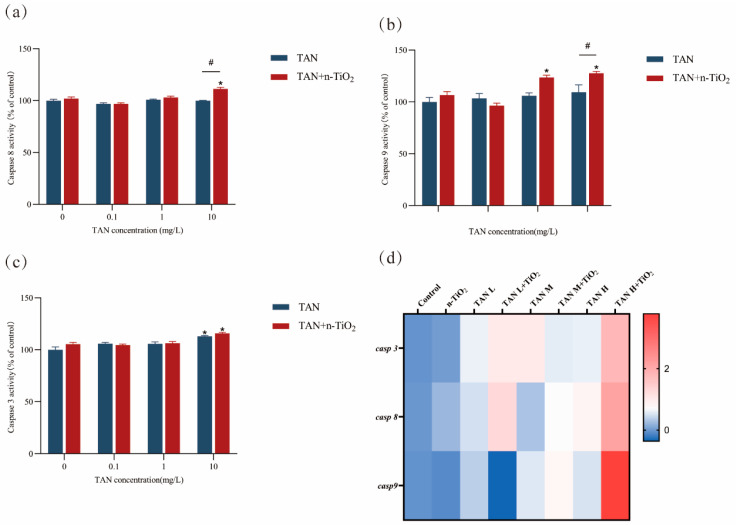
Analysis of caspase activity and apoptosis-related gene expression in zebrafish larvae following 120-hour exposure: (**a**) Caspase 8 activity; (**b**) Caspase 9 activity; (**c**) Caspase 3 activity; (**d**) heatmap of relevant gene expression, with values derived from log_2_-transformed fold changes in gene expression. Data are presented as mean ± SEM (n = 3 biological replicates); * indicates significant differences between control and exposed groups (*p* < 0.05); # indicates significant differences between TAN and TAN + n-TiO_2_ treatment groups (*p* < 0.05).

Results of caspase family activity assays (Figure 7) showed that, compared with the control group, exposure to n-TiO_2_ alone had no significant effect on Caspase 3, Caspase 8, and Caspase 9 activity. In the TAN-exposed group alone, Caspase 3 activity in the 10 mg/L group was significantly elevated compared to the control. In the 10 mg/L TAN + n-TiO_2_ group, Caspase 3, Caspase 8, and Caspase 9 activities were all significantly elevated, with Caspase 8 and Caspase 9 activities significantly higher than in the 10 mg/L TAN group (*p* < 0.05). Additionally, *casp3*, *casp8*, and *casp9* were significantly upregulated in the 10 mg/L TAN + n-TiO_2_ group. This suggested that combined exposure exacerbates neuronal apoptosis by activating both intrinsic and extrinsic apoptotic pathways.

### 3.8. Determination of AChE Activity and DA Content

**Figure 8:**
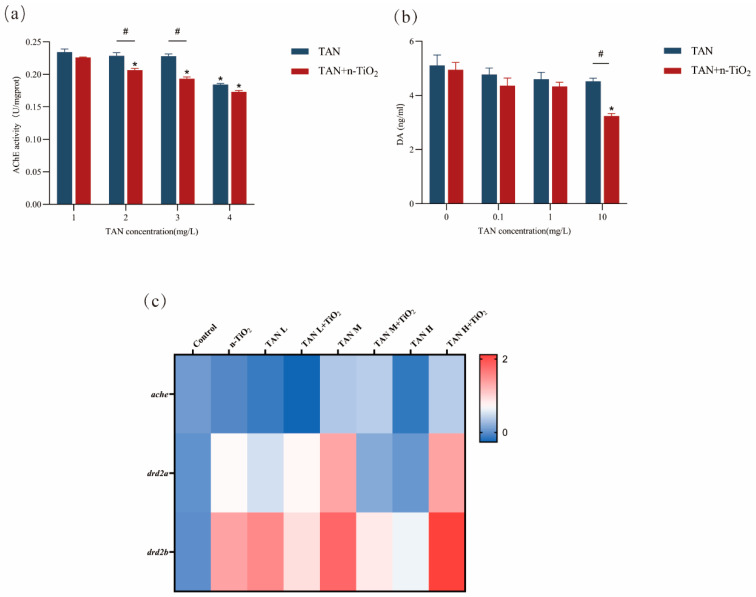
Analysis of AChE activity and DA content in zebrafish larvae following 120 h exposure: (**a**) AChE activity; (**b**) DA content; (**c**) heatmap of related gene expression levels, where values represent fold changes in gene expression log-transformed to base 2. Data are presented as mean ± SEM (n = 3 biological replicates); * indicates significant differences between control and exposed groups (*p* < 0.05); # indicates significant differences between TAN and TAN + n-TiO_2_ treatment groups (*p* < 0.05).

Neurotransmitter detection results (Figure 8) indicated that n-TiO_2_ exposure alone had no significant effect on DA content or AChE activity. Compared to the control group, AChE activity was significantly reduced in the 10 mg/L TAN group. AChE activity was significantly reduced in all combined exposure groups, with concentrations of 0.1 and 1 mg/L showing significantly lower AChE activity than their respective single-exposure counterparts. DA content was significantly reduced only in the 10 mg/L TAN + n-TiO_2_ group. Furthermore, compared to the single-exposure groups, gene expression levels of *drd2a* and *drd2b* were significantly upregulated.

### 3.9. Result of Gene Expression

**Figure 9:**
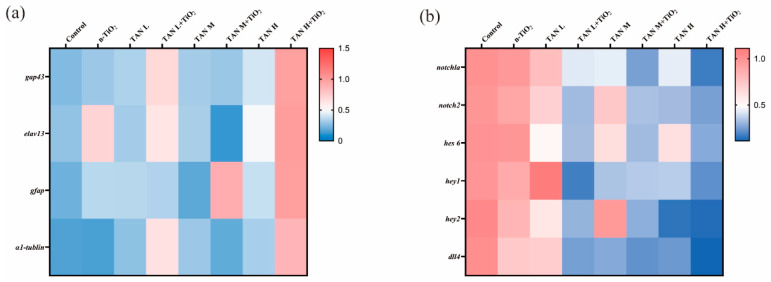
Analysis of neural development and Notch pathway gene expression in zebrafish larvae after 120 h exposure: (**a**) heatmap of neural development-associated gene expression; (**b**) heatmap of Notch signaling pathway gene expression. Data represent log_2_-transformed fold changes relative to control from three independent biological replicates. Values indicate fold changes in gene expression log-transformed to base 2. Data are presented as mean ± SEM (n = 3 biological replicates).

After 120 h of exposure, the expression levels of genes related to neural and retinal development in all groups of zebrafish larvae were detected (Figure 9a). Compared with the control group, the expression of *gap43*, *elavl3*, *gfap*, and *a1-tubulin* genes was significantly upregulated in the high-concentration combined exposure group (*p* < 0.05). Monitoring of Notch pathway-related genes showed (Figure 9b) that compared with the control group, the expression levels of *notch1a*, *notch2*, *hes6*, *hey1*, *hey2*, and *dll4* genes were significantly decreased in the 10 mg/L TAN group (*p* < 0.05), and the combined exposure exacerbated this effect.

### 3.10. Transcriptomic Analysis

**Figure 10:**
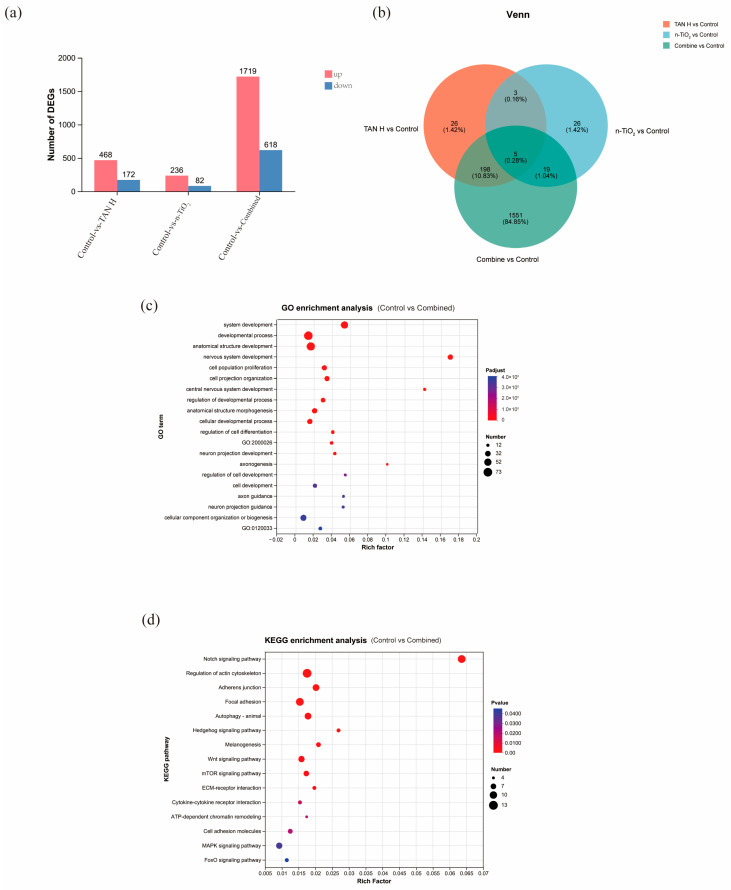
(**a**) Number of differentially expressed genes among different treatment groups; (**b**) Venn diagram of differentially expressed gene sets between each treatment group and the control group; (**c**) GO enrichment bubble chart; (**d**) KEGG enrichment bubble chart.

The transcriptomic analysis results are presented in Figure 10. Figure 10a displays the statistical distribution of differentially expressed genes across experimental groups. Compared with the control group, the co-exposure group exhibited 2337 differentially expressed genes, with 1719 being upregulated and 618 downregulated. Figure 10b presents the overlapping relationships of differentially expressed genes between each treatment group and the control group in Venn diagrams. Based on KEGG pathway annotation, we selected nervous system-related differentially expressed genes for further investigation. Systematic cluster analysis, GO functional annotation, and KEGG pathway enrichment analysis were conducted on the 74 selected nervous system-related differentially expressed genes. KEGG enrichment analysis indicates that TAN may affect nervous system function through the regulation of key signaling pathways, including Notch signaling, actin cytoskeleton reorganization, autophagy, Wnt signaling, and MAPK signaling pathways.

## 4. Discussion

This study systematically investigated developmental neurotoxicity induced by the co-exposure of n-TiO_2_ and TAN in zebrafish embryos. Our results showed that while n-TiO_2_ (100 µg/L) alone at an environmentally relevant concentration induced no overt toxicity, its co-presence with TAN significantly exacerbated TAN-induced neurotoxicity. This synergistic effect manifested as developmental impairment, behavioral abnormalities, increased apoptosis, oxidative stress imbalance, and disruption of neurotransmitter systems. Collectively, our findings highlight the significant ecological risk posed by n-TiO_2_ as a potent enhancer of TAN-induced neurodevelopmental toxicity in aquatic organisms.

In the present study, our results indicated that n-TiO_2_ significantly enhances TAN accumulation in zebrafish. n-TiO_2_ has been reported to act as a carrier for environmental pollutants, potentially influencing their bioaccumulation in aquatic organisms [21]. Whether n-TiO_2_ can enter biological systems depends on its ability to penetrate biological membranes [22]. Previous research reported that zebrafish villi measure approximately 600 nm in size [23]. This study measured the average particle size of n-TiO_2_ in aqueous solution to be 99.8 nm, suggesting potential for n-TiO_2_ to penetrate villi and accumulate within zebrafish. Therefore, it is reasonable to speculate that n-TiO_2_ may act as a carrier for TAN, leading to significant TAN accumulation in the larvae and causing severe health impacts.

Motor behavior has been widely recognized as a critical indicator for the effects of toxic substances on zebrafish neurobehavior [24]. In the present study, we assessed the swimming behavior of zebrafish larvae after 120 h of exposure. The results showed that zebrafish larvae exhibited progressively slower activity in the TAN exposure group. Furthermore, compared to TAN exposure alone, a greater reduction in activity was observed in the n-TiO_2_-co-TAN exposure group, suggesting n-TiO_2_ could exacerbate TAN-induced neurotoxicity. Short-term sublethal TAN exposure induced neurotoxicity, altering fish behavior by increasing fear responses and decreasing schooling cohesion [9]. Xu et al. [25] found significant declines in fish activity levels and vitality after one day of exposure to 100 mg/L TAN solution. Xing et al. [26] demonstrated that polystyrene nanoplastics (PSNPs) exacerbate TAN-induced cholinergic system disruption in zebrafish, thereby altering their motor behavior. In addition, n-TiO_2_ is known to enhance the toxicity of other pollutants through its carrier effect. Wu et al. [16] found that co-exposure with n-TiO_2_ further aggravated brain tissue damage by intensifying the oxidative stress levels induced by MCLR. Therefore, the significant attenuation of zebrafish motility in the combined TAN and n-TiO_2_ exposure group may reflect the exacerbation of TAN-induced neurotoxicity in zebrafish embryos by n-TiO_2_.

Behavioral alterations in zebrafish are typically closely associated with oxidative damage to brain tissue [27]. In this experiment, TAN exposure significantly increased MDA levels and decreased GSH content in zebrafish larvae brains, while reducing SOD and Gpx activity. Concurrently, n-TiO_2_ presence amplified these changes. Studies indicate that elevated TAN concentrations in aquatic environments induce reactive oxygen species (ROS) production in aquatic organisms [28]. Excessive ROS production disrupts the cerebral redox balance and induces lipid peroxidation by reacting with unsaturated fatty acids and cholesterol in cell membranes [29]. This process reduces membrane fluidity, increases permeability, and generates oxidative byproducts such as MDA. As a hallmark of lipid peroxidation, MDA can cross-link with nucleic acids, proteins, and nucleophospholipids via its nucleophilic groups, leading to cytotoxic effects upon accumulation [30]. To counteract oxidative stress, the antioxidant defense system eliminates excess MDA through both enzymatic and non-enzymatic mechanisms [21]. SOD primarily catalyzes the dismutation of superoxide radicals [31], while GPx acts as a detoxifying agent by reducing lipid hydroperoxides to their corresponding alcohols and converting hydrogen peroxide to water, simultaneously oxidizing glutathione [32]. As a key non-enzymatic antioxidant, GSH plays critical roles in antioxidant defense, detoxification, and metabolic regulation, making it a central component of the antioxidant system [33]. Under physiological conditions, *nrf2* binds to its inhibitor *keap1* in the cytoplasm and undergoes rapid degradation. Upon oxidative stress, *nrf2* dissociates from Keap1, translocates into the nucleus, binds to antioxidant response elements (AREs), and activates the transcription of various antioxidant and detoxification genes [34]. To further elucidate the mechanism of n-TiO_2_ exacerbating TAN-induced oxidative stress, we found that TAN exposure significantly induced the expression of the *keap1*, *nrf2*, and *gpx1a* genes. Notably, co-exposure to n-TiO_2_ markedly enhanced the upregulation of these genes. In conclusion, our findings provide compelling evidence that n-TiO_2_ exacerbates TAN-induced swimming abnormalities and neurotoxicity in zebrafish embryos by intensifying oxidative stress.

Apoptosis is a critically involved process in the developmental neurotoxicity of environmental pollutants [35]. As a form of programmed cell death, apoptosis undergoes precise and stringent regulation, playing an irreplaceable and pivotal role in the normal growth and development of organisms [36]. Previous research has proven that TAN could induce apoptosis in the organs of the dark-striped pufferfish [37]. In this experiment, AO staining results revealed that apoptotic cells were concentrated in neural tissues such as the brain and spinal cord, with stronger signals observed in the combined exposure group. To delineate the molecular mechanisms, we investigated the caspase-dependent pathways. *Caspase 8* and *caspase 9*, upon stimulation by cytochrome C, initiate a downstream caspase cascade. Once activated, Caspase 3 acts as the primary executor of apoptosis, enhancing its efficiency [38]. Lin et al. [39] found that TAN exposure elevated transcription levels of the apoptosis gene *casp3* in zebrafish embryos, thereby inducing keratinocyte apoptosis. In this study, both Caspase 3, Caspase 8, Caspase 9 enzyme activity and *casp3, casp8*, and *casp9* gene expression were significantly upregulated in the combined exposure group, indicating that n-TiO_2_ could exacerbate TAN-induced apoptosis through a caspase-dependent pathway.

Another potential mechanism for neurotoxicity induced by n-TiO_2_ and TAN involves cholinergic system disruption. During early life stages of zebrafish, AChE within the cholinergic system is essential for neuronal and muscular development [40]. In this study, we observed a concentration-dependent inhibition of AChE activity by TAN, which was significantly potentiated by n-TiO_2_ co-exposure. AChE catalyzes the hydrolysis of acetylcholine in the synaptic cleft, playing a crucial role in neural signaling. It serves as a biomarker for detecting developmental neurotoxicity and correlates with motor behavior; changes in its activity are important indicators for assessing neurotoxicity [41]. Xing et al. [26] demonstrated that exposure to PSNPs and TAN induces developmental neurotoxicity in zebrafish embryos by altering AChE activity and *ache* gene expression. Therefore, n-TiO_2_ may exacerbate TAN-induced neurotoxicity by altering AChE activity in zebrafish. Additionally, the mechanism by which combined exposure to n-TiO_2_ and TAN induces neurotoxicity in zebrafish may also involve alterations in dopaminergic neurons [42]. Previous studies reported that dopamine plays an important role in motor control, cognition, and motivation, particularly in the development of dopaminergic neurons and circuits [43,44]. In the present study, we found that dopamine levels and gene expression of related genes (*drd2a* and *drd2b*) in zebrafish changed progressively following TAN exposure, with n-TiO_2_ exacerbating this trend. The synergistic disruption of both cholinergic and dopaminergic systems by n-TiO_2_ and TAN offers a physiological basis for the severe neurobehavioral deficits observed.

Given the close association between early swimming behavior and nervous system development in zebrafish, this study analyzed the expression of central nervous system-related genes (*gap43*, *elavl3, gfap*, and *α1-tubulin*). A marked dysregulation of these genes was observed, which was significantly amplified in the co-exposure group. Functionally, these genes can be categorized into three groups: *gap43* and *α1-tubulin* are involved in axonal growth and neuronal cytoskeleton assembly, regulating axonal regeneration and microtubule formation, respectively [45,46,47]; *elavl3* serves as a key marker gene for nervous system development, maintaining normal neuronal function [48,49]; and *gfap* reflects the cytoskeletal integrity of astrocytes [50,51]. Previous studies have shown that upregulation of axon-related genes following tris (1,3-dichloroisopropyl) phosphate and dechlorane plus exposure often represents a compensatory response to axonal growth inhibition [52,53]. The gene expression patterns observed in this experiment are consistent with this phenomenon, suggesting that the upregulation is likely a compensatory mechanism for impaired neuronal axon development. Notably, n-TiO_2_ co-exposure intensified this dysregulation, which strongly suggests a more severe underlying damage to neuronal development. Therefore, we conclude that the synergistic disruption of these critical neurodevelopmental processes, from axonal growth to astrocyte function, constitutes a key molecular mechanism underlying the exacerbated behavioral abnormalities. This mechanism is likely driven by the previously demonstrated role of n-TiO_2_ in enhancing TAN bioaccumulation, leading to more profound neurodevelopmental damage.

To systematically elucidate the neurotoxic mechanisms of ammonia in zebrafish, this study conducted an in-depth analysis using transcriptome sequencing technology. Based on the screened nervous system-related differentially expressed genes, we performed systematic cluster analysis and functional enrichment analysis. The results showed significant alterations in neuronal development-related pathways as well as oxidative stress and apoptosis-related pathways. Existing studies reported that the Wnt/β-catenin signaling pathway plays a crucial role in key biological processes, including neural development, cell survival, cell cycle regulation, and microglial activation [54]. This signaling pathway possesses multidimensional core regulatory functions in nervous system development, functional maintenance, and neural repair. Our findings suggest that n-TiO_2_ and TAN may affect nervous system development by modulating this pathway. As an important stress response mechanism in eukaryotic cells, autophagy achieves degradation and recycling of organelles and proteins through the lysosomal pathway. Research demonstrated that during neural development and neuronal homeostasis maintenance, autophagy participates in regulating key physiological processes, including presynaptic function, synaptic remodeling, and synaptic plasticity [55,56]. The MAPK signaling pathway, serving as a core regulatory pathway for neurophysiological function and pathological damage, has been confirmed as a common target for various neurotoxic substances (including mycotoxins and environmental pollutants) [57]. Abnormal activation or inhibition of this pathway disrupts the dynamic balance between neuronal apoptosis and survival, ultimately leading to neuronal damage. Our findings indicate that n-TiO_2_ and TAN may produce neurotoxic effects by synergistically affecting these key signaling pathways, particularly through interfering with neuronal development processes and stress response mechanisms.

To validate our transcriptomic findings, we quantified the expression of key genes within the Notch signaling pathway (*notch1a*, *notch2*, *hes6*, *hey1*, *hey2*, and *dll4*). The Notch signaling pathway represents a central regulatory pathway in the development of multiple organ systems, including hematopoiesis, angiogenesis, and neurogenesis [58]. Previous studies have demonstrated that Notch activation downregulates *Xic1* gene transcription and inhibits primordial neuron formation [59]. Furthermore, Notch has been shown to regulate apoptosis in early neural progenitor cells [60]. Despite its pivotal role, a potential connection between TAN-induced neurodevelopmental defects and Notch signaling had not been established. Our qPCR results demonstrated that TAN exposure suppresses the Notch signaling pathway, an effect that was significantly potentiated by n-TiO_2_ co-exposure. This identifies the Notch pathway as a novel and previously unrecognized target for TAN-mediated neurodevelopmental toxicity. Nevertheless, the precise molecular initiation event represents an important focus for future research.

## 5. Conclusions

In conclusion, this study demonstrates that n-TiO_2_ acts as a critical enhancer of TAN-induced developmental neurotoxicity in zebrafish. The exacerbated toxicity is driven by a multi-mechanistic synergy, wherein n-TiO_2_ promotes the bioaccumulation of TAN, which in turn leads to severe oxidative stress, amplified neuronal apoptosis, and concurrent disruption of the cholinergic and dopaminergic systems, ultimately manifesting as impaired neural development and locomotor deficits. Future research should focus on elucidating the molecular mechanisms underlying the synergistic interaction between TAN and n-TiO_2_, exploring effective environmental monitoring and remediation strategies to mitigate the potential risks posed by these two pollutants to aquatic ecosystems and human health. Concurrently, studies on the combined toxicity of other common pollutants should be intensified to provide a more robust scientific basis for comprehensively assessing the ecological and health risks of environmental contaminants.

## Data Availability

The datasets used and/or analyzed during the current study are available from the corresponding author upon reasonable request.

## Supplementary Materials

The following supporting information can be downloaded at: https://www.mdpi.com/article/10.3390/toxics13121031/s1, Figure S1: larval distribution pattern; Table S1: Gene primer sequence.
