# Supplementary material for: Titanium Dioxide Nanoparticles Aggravated the Developmental Neurotoxicity of Ammonia Nitrogen on Zebrafish Embryos
Source: Toxics. 2025 Nov 28;13(12):1031. doi: 10.3390/toxics13121031 (PMC12737239; doi:10.3390/toxics13121031)

Titanium Dioxide Nanoparticles Aggravated the Developmental Neurotoxicity of Ammonia Nitrogen on Zebrafish Embryos

Minglei Lyu[1#], Jiaqian Yu[1#], Qing Yang[2*], Yi Shen[1], Haoling Liu[1], Xuanjie Wang[1], Xiaolin Liu[1],Fang Shi[2], Xi Zhou[2], Jinmiao Zha[3], Guangyu Li[1], Xufa Ma[1]

[1]College of Fisheries, Huazhong Agricultural University, Wuhan, 430070, PR China
[2]Key Laboratory of Ecological Impacts of Hydraulic Projects and Restoration of Aquatic Ecosystem of Ministry of Water Resources and Chinese Academy of Sciences, Wuhan, 430079, PR China
[3]National Engineering Research Center of Industrial Wastewater Detoxication and Resource Recovery, Research Center for Eco-Environmental Sciences, Chinese Academy of Sciences, Beijing 100085, PR China
[#]These two authors contribute equally to this work
*Corresponding Author:
Qing Yang
Key Laboratory of Ecological Impacts of Hydraulic Projects and Restoration of Aquatic Ecosystem of Ministry of Water Resources and Chinese Academy of Sciences, Wuhan, 430079, PR China
Email: yangqinghust@hust.edu.cn

Table S1. Gene primer sequence

Gene name	Primer sequences (from 5′ to 3′)
ache-F	CCCTCCAGTGGGTACAAGAA
ache-R	GGGCCTCATCAAAGGTAACA
gap43-F	TGCTGCATCAGAAGAACTAA
gap43-R	CCTCCGGTTTGATTCCATC
elavl3-F	AGACAAGATCACAGGCCAGAGCTT
elavl3-R	TGGTCTGCAGTTTGAGACCGTTGA
gapdh-F	CTGGTGACCCGTGCTGCTT
gapdh-R	TTTGCCGCCTTCTGCCTTA
gfap -F	GGATGCAGCCAATCGTAAT
gfap -R	TTCCAGGTCACAGGTCAG
α1-tublin-F	AATCACCAATGCTTGCTTCGAGCC
α1-tublin-R	TTCACGTCTTTGGGTACCACGTCA
drd2b-F	AGCCCCCTCTCAAGGAAAAG
drd2b-R	GTACACCCCCGTTGGTCTTA
drd2a	TGGTACTCCGGAAA AGACG
drd2a	ATCGGGATGGGTGCATTTC
Notch1a-F	GCCCGGATGGTCAGGTAAAA
Notch1a-R	ATGGAAGTTCGGAGGACACG
Notch2-F	ATGATGCCACCTGCTTGGAT
Notch2-R	TTCCCCAGTGAAACCCTGAG
Dll4-F	ACTACCAGGTGGTCGTGTCT
Dll4-R	ATCAATGAAAATGACCCCGGC
Hey1-F	TGAGGATACGCGCTGCTAAA
Hey1-R	GAGAATTCGCACCGGCATTT
Hey2-F	GGCCACAGGAGGAAAAGGATATT
Hey2-R	GCTGCTGAGGTGAGAAACCA
Hes6-F	TTGTCTCGAACTGAACCCGC
Hes6-R	CCAGCGGTTTCCTCGTCTTT

nrf2-R	CCCGGTGAGAAGCTCTGTAG
nrf2-F	TGGCCCTGAAGAATTTAACG
keap1-F	TGATGGACAAACCCAACTCA
keap1-R	CACTGGACAGGAAACCACCT
gpx1a-F	AGGCACAACAGTCAGGGATT
gpx1a-R	CAGGAACGCAAACAGAGGG

Figure S1. larval distribu-tion pattern

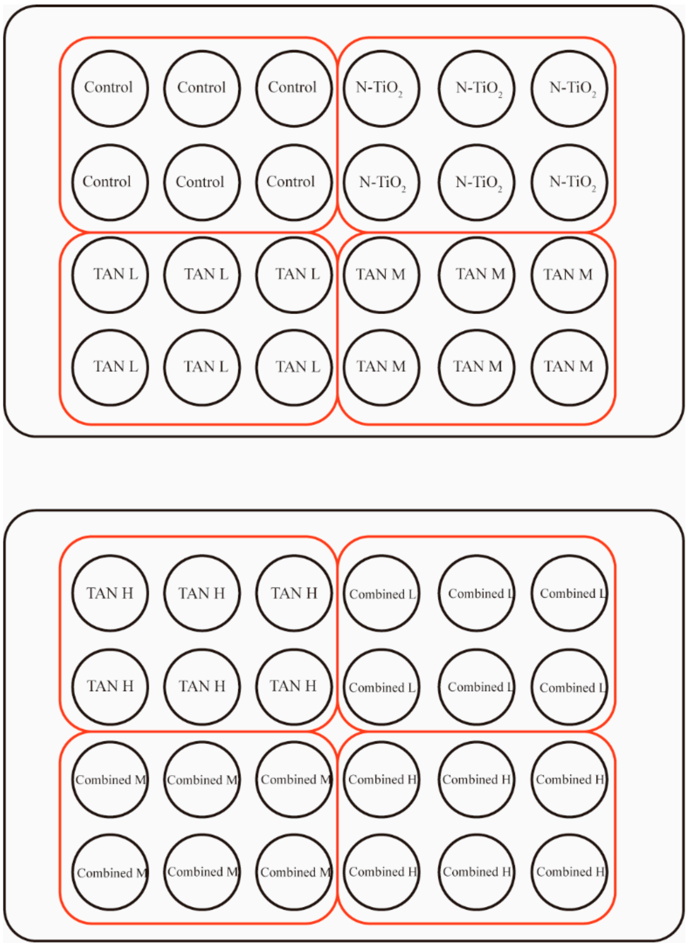

Supplement: Supplementary file 1 [file toxics-13-01031-s001.zip › toxics-3985127-supplementary.pdf]
